# Comparing Literature- and Subreddit-Derived Laboratory Values in Polycystic Ovary Syndrome (PCOS): Validation of Clinical Data Posted on PCOS Reddit Forums

**DOI:** 10.2196/44810

**Published:** 2023-08-25

**Authors:** Rebecca H K Emanuel, Paul D Docherty, Helen Lunt, Rebecca E Campbell

**Affiliations:** 1 Department of Mechanical Engineering University of Canterbury Christchurch New Zealand; 2 Diabetes Services Te Whatu Ora Waitaha Canterbury Canterbury New Zealand; 3 Department of Medicine University of Otago Canterbury New Zealand; 4 Department of Physiology, School of Biomedical Sciences Centre for Neuroendocrinology University of Otago Dunedin New Zealand

**Keywords:** androgens, clinical treatment, cohort, laboratory tests, medical intervention, metabolic markers, online forum, ovary, PCOS, polycystic ovary syndrome, reddit, reproductive hormones, reproductive, social media, validation study

## Abstract

**Background:**

Polycystic ovary syndrome (PCOS) is a heterogeneous condition that affects 4% to 21% of people with ovaries. Inaccessibility or dissatisfaction with clinical treatment for PCOS has led to some individuals with the condition discussing their experiences in specialized web-based forums.

**Objective:**

This study explores the feasibility of using such web-based forums for clinical research purposes by gathering and analyzing laboratory test results posted in an active PCOS forum, specifically the PCOS subreddit hosted on Reddit.

**Methods:**

We gathered around 45,000 posts from the PCOS subreddit. A random subset of 5000 posts was manually read, and the presence of laboratory test results was labeled. These labeled posts were used to train a machine learning model to identify which of the remaining posts contained laboratory results. The laboratory results were extracted manually from the identified posts. These self-reported laboratory test results were compared with values in the published literature to assess whether the results were concordant with researcher-published values for PCOS cohorts. A total of 10 papers were chosen to represent published PCOS literature, with selection criteria including the Rotterdam diagnostic criteria for PCOS, a publication date within the last 20 years, and at least 50 participants with PCOS.

**Results:**

Overall, the general trends observed in the laboratory test results from the PCOS web-based forum were consistent with clinically reported PCOS. A number of results, such as follicle stimulating hormone, fasting insulin, and anti-Mullerian hormone, were concordant with published values for patients with PCOS. The high consistency of these results among the literature and when compared to the subreddit suggests that follicle stimulating hormone, fasting insulin, and anti-Mullerian hormone are more consistent across PCOS phenotypes than other test results. Some results, such as testosterone, sex hormone–binding globulin, and homeostasis model assessment–estimated insulin resistance index, were between those of PCOS literature values and normal values, as defined by clinical testing limits. Interestingly, other results, including dehydroepiandrosterone sulfate, luteinizing hormone, and fasting glucose, appeared to be slightly more dysregulated than those reported in the literature.

**Conclusions:**

The differences between the forum-posted results and those published in the literature may be due to the selection process in clinical studies and the possibility that the forum disproportionally describes PCOS phenotypes that are less likely to be alleviated with medical intervention. However, the degree of concordance in most laboratory test values implied that the PCOS web-based forum participants were representative of research-identified PCOS cohorts. This validation of the PCOS subreddit grants the possibility for more research into the contents of the subreddit and the idea of undertaking similar research using the contents of other medical internet forums.

## Introduction

### Polycystic Ovary Syndrome

Polycystic ovary syndrome (PCOS) is a condition that affects 4% to 21% of reproductive-aged people with ovaries [1]. PCOS is diagnosed most commonly by the Rotterdam criteria [2], with concurrent presentation of 2 or 3 of the following: evidence of biochemical or clinical hyperandrogenism, evidence of anovulation or oglio-ovulation, and ultrasound evidence of polycystic ovarian morphology [3]. As such, PCOS is a heterogenous condition [4]. PCOS is a diagnosis of exclusion, usually requiring an absence of thyroid disease, nonclassic congenital adrenal hyperplasia, and hyperprolactinemia [5]. Cushing syndrome and adrenal tumors are additional common exclusions [5]. Although PCOS was formally defined as early as 1935 [6], internationally accepted evidence-based guidelines for the assessment and management of PCOS were published as late as 2018 [2]. However, the evidence base available to support treatment guidelines was rated moderate to low by its internal assessment criteria [2].

As a heterogeneous condition, PCOS has a variety of associated presentations and possible etiologies [3]. Thus, characterizing the distinct phenotypes of PCOS is very difficult in small-scale studies. This limitation can be mitigated if recruitment criteria focus on a specific possible phenotype of PCOS. It is difficult to obtain suitably large PCOS data sets using conventional clinical study designs due to the cost and administrative burden of large-scale studies. Thus, alternative methods of obtaining large PCOS data sets could be useful.

### The State of PCOS Management and Diagnosis

Several surveys of clinicians and patients have been carried out to assess the state of PCOS management and diagnosis. Clinicians have expressed concerns about limited PCOS treatment options and their lack of supporting evidence [7], uncertainty regarding diagnosis and management [8], the need for more tailored care toward individuals with PCOS [8], and a hesitance to precisely follow the Rotterdam diagnostic criteria due to overdiagnosis concerns [9]. A large-scale survey showed that of the individuals who meet the Rotterdam criteria, those with lower BMI are less likely to receive a PCOS diagnosis [10]. Patients with PCOS with lower BMIs or without all the Rotterdam criteria are also less likely to be referred to clinical studies of PCOS [11]. These factors could lead to the neglect of certain PCOS phenotypes in the literature.

Surveys of patients with PCOS revealed widespread dissatisfaction with traditional treatment options such as oral contraceptives and ovulation-inducing agents [7,12]. This was often due to distressing side effects and minimal symptom improvement [7]. Surveyed people with PCOS also expressed dissatisfaction with their physicians’ awareness of PCOS [13,14] and the lack of information provided to them about PCOS or relevant clinical therapies upon diagnosis [15].

### The PCOS Reddit Forum

Some people with PCOS find and participate in online PCOS support groups that exist on various social media platforms. This is possibly due to dissatisfaction with clinical diagnosis and management options. Online support groups can contain written accounts of the challenges of living with PCOS from a cohort that is larger than most clinical studies [16]. A survey indicated that people with PCOS regard online PCOS support groups as helpful for learning how to manage PCOS [17]. However, some found it distressing to read about other people’s hardships or felt more isolated after reading about others experiences that did not match their own [17].

In this study, laboratory test results posted to a publicly available online PCOS support group were collected to explore the feasibility of using information posted to an online support group to investigate PCOS. This was done using a mixture of manual processing and machine learning methods. The PCOS subreddit [16], hosted on Reddit, was chosen due to its large number of participants (approximately 30,000), public availability of posts and comments, and encouragement from the platform curators to access and use the data.

### The Potential of Reddit and Machine Learning Research

The content of the PCOS subreddit could yield a large volume of data exploring people’s experiences with PCOS, the symptoms they present with, the treatments they use, and their perceived treatment outcomes. This kind of large data set could prove useful for studying heterogeneous conditions that require a large number of participants to compare and contrast phenotypes. However, processing the subreddit has its own limitations. The biases seen within the subreddit are different from those seen in traditional research due to the self-reported nature of the data and the requirements to participate in the subreddit. The large volume of data leads to significant data extraction challenges. Manual approaches to reading and interpreting data may be too arduous to undertake with sufficient consistency. Hence, automated machine learning approaches were also implemented in this study. However, any automated approach would be subject to errors and misclassification. Thus, to establish the PCOS subreddit forum as a suitable data source for PCOS research, the relevance and validity of the extracted data must be assessed.

Previously, self-reported accounts have been used to discuss PCOS [10,18,19], and Reddit data has been used in clinical research [20-22]. However, to the authors’ knowledge, no studies have directly processed the contents of an online PCOS support group. This paper validates the research potential of the PCOS subreddit data set by comparing the laboratory test results found in the subreddit to the laboratory results of 10 peer-reviewed PCOS studies. The decision was made to assess the validity of the subreddit data through laboratory results, as they are an easy metric to compare with clinical laboratory data reported in research studies using statistical methods. Once the data extraction methods and content of the subreddit have been validated with the comparison of laboratory results, the discussions of symptoms and treatment methods within the subreddit can be explored in future research.

## Methods

### Data Collection and Cleaning

On the May 3, 2021, all historic posts and comments were gathered from the web-based PCOS subreddit [16] using the Pushshift Reddit Dataset [23]. No recruiting or direct interaction with the PCOS subreddit was carried out as part of the data collection. The Pushshift Reddit Dataset is an archive of all platform content since 2015 and is intended for research use [23]. Reddit is organized into posts and their replies, called comments. Users can reply to comments, leading to multifurcating discussion threads. This PCOS subreddit data set contained a total of approximately 45,000 posts and 300,000 comments from 30,000 unique accounts [16]. Only posts were used in laboratory test analysis. The posts were randomly shuffled, and the first 5000 of the shuffled posts were separated for manual processing. These posts were read and labeled to identify the presence of laboratory test results. Table 1 contains a summary of the abbreviations and units used for the laboratory test results and other user information explored in this study.

In order to process the remaining 40,000 posts, machine learning was used to select relevant posts. Global vectors word embeddings [24], a method for translating words into vectors, were created with the text from all PCOS subreddit posts and comments for use in machine learning. Using the labeled posts and global vectors embeddings, a convolutional neural network (CNN), a type of machine learning, was trained to indicate whether a text post may contain laboratory test results. A subset of the labeled posts were not used in training and were instead used to approximate the accuracy of the network. CNN structure and training are described in Multimedia Appendix 1 [25,26].

The posts likely to contain laboratory test results were manually read, and relevant results were recorded. Ages, BMIs, or information pertaining to menstrual cycle phase were also recorded when available. Ages were sometimes approximated using the dates that posts were created and other posts from the same account in which they stated their age. BMIs were typically calculated using given height and weight information. As results were recorded, subreddit values were compared to standard laboratory test result ranges [27] to ensure values were not nonsense values or mistranscribed units. Units were only considered mistranscribed if the given unit was not a common unit for the given test, the value and unit combination did not make physiological sense, or the value made significantly more physiological sense if the unit was a different common unit. The manner in which PCOS may affect results was considered during this process. For example, an abnormally high total testosterone (total T) result of 90 ng/dL (healthy reference range [20-75 ng/dL]) was considered reasonable for PCOS, while dehydroepiandrosterone sulfate (DHEA-S) of 8 g/dL was considered a possible unit mistranscription (healthy reference range [59-328 g/dL] or [1.6-8.9 mol/L]). Table S1 in Multimedia Appendix 2 shows a table of specific considerations for possible unit or value mistranscription. Cases where units were considered mistranscribed were exceedingly rare.

**Figure 1:**
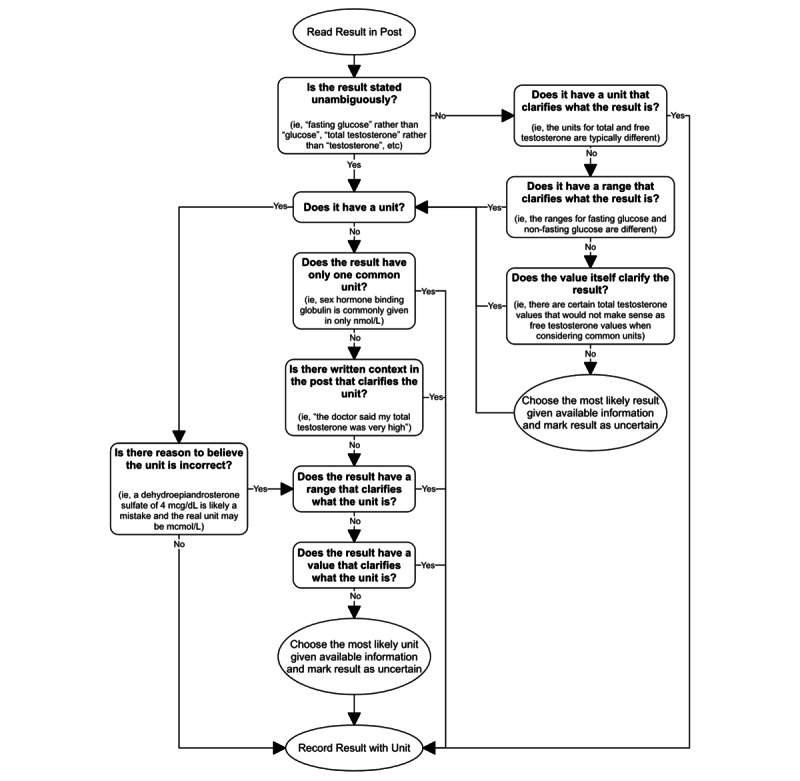
The test result recording process. Ellipses represent actions, and boxes represent decisions.

In some cases, the laboratory test results were presented with full, unambiguous names, units, and ranges. In other cases, the results were written without units or the full test name. Typically, the test result and correct unit could be determined with the given information. If either were unclear, the result was marked as uncertain. Figure 1 shows the test recording process and when a result would be marked as uncertain. When all results were recorded, unit conversions were made so that each test result was recorded with a single unit. Unit conversions are shown in Multimedia Appendix 3.

Some posts with laboratory test results were attached to the same account or contained multiple sets of test results from different time periods. Between tests, significant lifestyle changes or new treatments were often applied, leading to the same person presenting with very different results and symptoms. Due to these drastic changes in PCOS presentation, each set of tests taken on the same day was considered its own point of data, separate from other tests taken from the same person on different days. Given the nature of the data, knowing what medications people were taking at the time of the laboratory tests was not possible.

Once all the laboratory test results were gathered, some reprocessing was carried out to maximize accuracy. All results marked as uncertain and results with an absolute *z* score greater than 2 were reprocessed. Occasionally, a person would discuss the same test results in different posts. To prevent a single result from appearing multiple times, all identical results by the same account were analyzed and duplicate results were omitted from further analysis, even if they were posted across multiple days. Given the results from a particular day of testing, some accounts reported portions of the results across multiple posts. In obvious cases of this, the results were merged into 1 set.

Some laboratory test results were numerically extreme when compared to other results. Since gathering data from the internet is noisy by nature, mistranscriptions and outliers were to be expected. Extreme outlier removal seemed necessary before further processing could be undertaken. Any test result with an absolute *z* score greater than 4 was removed. While a *z* score of 2 or 3 for outlier removal is more established [28], characterizing disease states that are known for outlier behavior should be undertaken with a greater allowance for outliers than is typical. The rate of outlier removal was calculated for each test. The frequency with which different laboratory test results, ages, BMIs, and pieces of menstrual cycle information appeared within the data set varied. Summary statistics were derived for each type of value that appeared in the data at least 20 times. Percentages were calculated for the frequencies of each type of value among all the data and the proportion of uncertain values for each laboratory test.

**Table 1:** Relevant context of literature polycystic ovary syndrome (PCOS) populations used in comparisons.

Abbreviation	Result description	Unit used in this study
Age	Age at time of laboratory test	Years
Total T	Total testosterone	ng/dL
DHEA-S	Dehydroepiandrosterone sulfate	μg/dL
BMI	BMI at time of laboratory test	kg/m^2^
LH/FSH	Ratio of luteinizing hormone to follicle stimulating hormone	—^a^
Free T	Free testosterone	pg/mL
FPG	Fasting plasma glucose	mg/dL
FSH	Follicle stimulating hormone	U/L
HbA_1c_	Glycated hemoglobin	%
LH	Luteinizing hormone	U/L
TSH	Thyroid stimulating hormone	mU/L
PRL	Prolactin	ng/mL
E2	Estradiol	pg/mL
FI	Fasting insulin	mU/L
P	Progesterone	ng/mL
SHBG	Sex hormone binding globulin	nmol/L
AMH	Anti-Mullerian hormone	ng/mL
HOMA-IR	Homeostasis Model Assessment-Estimated Insulin Resistance Index (calculated using fasting insulin and fasting plasma glucose)	—
FT4	Free thyroxine	ng/dL
17-OHP	17-hydroxyprogesterone	ng/dL
Vit D	Vitamin D	ng/mL
FER	Ferritin	ng/mL
TC	Total cholesterol	mg/dL
LDL	Low-density-lipoprotein cholesterol	mg/dL
TG	Triglycerides	mg/dL
A4	Androstenedione	ng/dL
ALT	Alanine transaminase	U/L
HDL	High-density-lipoprotein cholesterol	mg/dL

^a^Not applicable.

### Relevant Literature Used

To explore how well the subreddit data set captured a PCOS population viable for research, comparisons were made between the PCOS subreddit data distributions and distributions from the aggregate of up to 10 relevant PCOS studies. Comparisons were also made between the subreddit and the individual studies. Test results with few values in the subreddit data or no strong relationship to PCOS were excluded from the comparison.

PCOS populations from 10 papers were chosen to represent the literature’s values. A search for peer-reviewed research relating to PCOS was carried out to identify papers reporting laboratory results from people with PCOS. Approximately 50 potentially useful papers were identified by prioritizing larger cohorts and a diverse range of countries and study contexts. Papers with fewer than 50 participants with PCOS in the study were excluded. It was also required that papers be less than 20 years old, allow for at least 5 relevant laboratory test comparisons, and had diagnosed their PCOS population through the Rotterdam criteria. All selected papers made exclusions for thyroid disease, nonclassic congenital adrenal hyperplasia, and hyperprolactinemia as part of their diagnosis. These criteria led to 10 papers for comparison. Critically, comparisons between the candidate papers and the subreddit data were not made before the papers were selected and had no bearing on which papers were used in this study. PCOS population sizes and other relevant information pertaining to the selected papers are shown in Table 2.

**Table 2:** Relevant context of literature polycystic ovary syndrome (PCOS) populations used in comparisons.

	PCOS group size, n	Cycle phase (when ovulatory)	Fasting status	Described ethnicity	Study type	Study context	Potential biases and specific differences to subreddit data set
Diamanti-Kandarakis and Panidis [29]	634	Follicular	Fasting	Greek Caucasian	Prospective	Exploring phenotypes within PCOS.	Only individuals in good health with no chronic or acute diseases and no medication that may affect results were included.
Cai et al [30]	600	Early Follicular	Fasting	Chinese	Cross-sectional observational	Exploring TSH^a^ levels in PCOS.	Smokers were excluded.
Tosi et al [31]	375	Not Specified	Fasting	97.1 % Caucasian (3.9% undeclared) (Italian Study)	Retrospective analysis	Exploring how well insulin resistance (IR) indexes capture IR in PCOS.	Tosi et al [26] identify a potential referral bias toward insulin resistant women. Women with diseases or treatments that affect IR were excluded.
Paschou et al [32]	372	Early Follicular	Fasting	Greek Caucasian	Cross-sectional observational	Investigating impact of adrenal hyperandrogenism on IR and lipid profile in PCOS.	Participants with additional medical illnesses, psychiatric illnesses, or treatments that would impact study were excluded.
Sova et al [33]	319	Early Follicular	Fasting	Nordic Caucasian	Cross-sectional observational	Exploring AMH^b^ levels in women with PCOS.	Only women with PCOS referred with anovulatory infertility and PCOM^c^. Exclusion of T2DM^d^, liver disease, history of cardiac or renal failure, hormonal treatment, smokers, or alcohol use.
Kumar et al [34]	213	Follicular	Fasting	12.7 % Black, 87.3 % White (Study in the United States)	Cross-sectional observational	Exploring presence of hyperandrogenism in PCOS.	None were apparent.
Bahceci et al [35]	98	Follicular	Fasting	Not specified (Turkish Study)	Cross-sectional observational	Exploring relationship between PCOS prolactin levels and IR.	Only normal BMI (20-25) women with PCOS without acanthosis nigricans.
Homburg et al [36]	90	Early Follicular	Not Specified	Not specified (English Study)	Prospective	Exploring relationship between AMH, PCOS and PCOM.	Only women with PCOS who sought out infertility treatment.
Pigny et al [37]	73	Early Follicular	Not Specified	Not specified (French Study)	Cross-sectional observational	Exploring if AMH levels could surrogate antral follicle count in PCOS diagnosis.	None were apparent.
Wright et al [38]	69	Early Follicular	Fasting	Not specified (Turkish Study)	Cross-sectional observational	Exploring effect of PCOS caused hormonal and metabolic abnormalities on thyroid.	Excluded smokers and patients with obesity, prediabetes, diabetes, severe iodine deficiency or medication that may affect results.

^a^TSH: thyroid stimulating hormone.

^b^AMH: anti-Mullerian hormone.

^c^PCOM: polycystic ovarian morphology.

^d^T2DM: type 2 diabetes mellitus.

### Statistical Analysis

To make comparisons, the means and SD were obtained or calculated for each paper. The aggregate means, SD, and numbers of people with PCOS were calculated using all 10 papers. Equivalence tests were performed using the two one-sided *t* test (TOST) procedure. An equivalence test is designed with the null hypothesis that the samples are significantly different. Hence, rejecting the null hypothesis of a TOST equivalence test allows us to conclude that there are no significant differences between the 2 samples [39]. Boundaries (*b*) are used in a TOST equivalence test to allow for nonsignificant differences between the samples [40]. A medium affect Cohen *d* value (±0.499) and the pooled SD from the groups being compared (*SD_pooled_*) were used to calculate the boundaries in the TOST tests (Equation 1) [40].



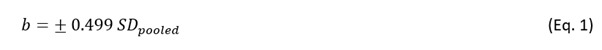



When equivalence was not confirmed, 1-sided *t* tests were performed to determine if a PCOS subreddit data mean was statistically higher or lower than a literature value.

### Ethical Considerations

An increasing portion of health sciences research uses data sets obtained from publicly accessible platforms such as social media websites, including Reddit [41]. The PCOS subreddit exploration was reviewed by the University of Canterbury (New Zealand) human ethics committee. As it fulfilled Australasian Human Research Ethics Consultancy Services (AHRECS) standards [42], it was considered by this committee to be low risk and out-of-scope. However, best practices regarding the ethical use of third-party consumer-based data sets and other research involving human participants in this setting are evolving [41,43].

The authors consider that their research fulfills current ethical guidelines for several reasons. First, regarding consent, the Reddit privacy policy for consumers of its services states:

Reddit also allows third parties to access public Reddit content via the Reddit API and other similar technologies [….] and you should take that into consideration before posting to the Services [44].

Furthermore, one of the deliberate intentions of Pushshift, the site where the data was retrieved, is to make the data on Reddit available for research such as this [23]. Second, this study is for public good rather than for reasons such as commercial gain. Third, the methodology behind the way Reddit data is used for research, for example, data cleaning methods, is transparently discussed in our methods section. Furthermore, Reddit users are anonymous and voluntarily publish their information. It was possible for a family member or friend to post on behalf of another. In all such cases, the data were not included. Care was also taken not to publicize any identifiable usernames. Fourth, the potential for bias is acknowledged. Finally, the researchers did not interact in any way with participants, for example, by using the subreddit to direct the generation of new information for the purposes of their research. Overall, the ethical risk of using this data set was thoroughly assessed and considered extremely low risk from a participant perspective.

## Results

### Data Collection and Cleaning

After training on 272 subreddit posts, CNN achieved an overall accuracy of 98% (89/91) on the testing data set (n=91) that contained examples of posts with and without laboratory test results. It achieved an accuracy of 96% (4429/4637) on a much larger testing data set (n=4637) including only posts without laboratory test results. These results imply the CNN is highly accurate but may be susceptible to some false-positive classifications. The trained CNN identified 3454 posts that were likely to contain laboratory test results in the approximate 45,000-post data set.

**Figure 2:**
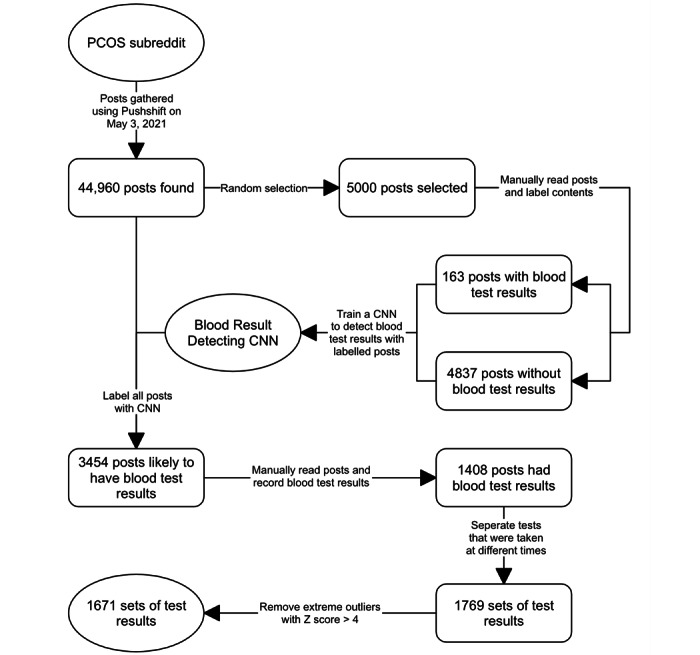
Diagram showing the gathering of the data set. Ellipses represent resources or tools. Boxes represent different stages of the processing. CNN: convolutional neural network; PCOS: polycystic ovary syndrome.

After the laboratory test results were extracted using the process in Figure 1, there were 1585 sets of laboratory test results and 6487 individual results in the data set. Figure 2 shows the process of retrieving the data set, and Table 3 is a summary of the final data set. Table S3 in Multimedia Appendix 4 provides the available data describing the cycle phase at the time of the laboratory testing. It is important to note that many of the tests taken in the luteal phase were done because of irregular cycles. Plots of ages and BMIs within the data set are given in Multimedia Appendix 5.

**Table 3:** Summary of data collected from the polycystic ovary syndrome (PCOS) subreddit of laboratory tests and other relevant information with more than 20 appearances.

	Number (no outliers), n	Portion of test sets with result (no outliers), %	Mean (SD) (no outliers)	Median (IQR) (no outliers)	Portion of results with uncertainty (with outliers), %	Outliers removed, %
Age (years)	661	41.7	25.5 (5.3)	24 (13-44)	0	0
Total T^a^ (ng/dL)	552	34.8	55.9 (37.9)	50 (1.5-375.1)	2.2	0.5
DHEA-S^b^ ( g/dL)	381	24	396.5 (165.2)	400 (15-1046)	0.8	1
BMI (kg/m^2^)	240	15.1	24.4 (6.4)	22.4 (15.4-48.6)	0	0
LH/FSH^c^	236	14.9	2.2 (1.4)	1.9 (0.1-7.9)	0.4	0.4
Free T^d^ (pg/mL)	226	14.3	5.9 (4.4)	4.9 (0.1-30)	6.6	1.3
FPG^e^ (mg/dL)	219	13.8	94.2 (17.6)	93 (50-157)	0.9	0.9
FSH^f^ (U/L)	214	13.5	5.8 (3.1)	5.5 (0.6-26.9)	0.5	0.5
HbA_1c_^g^ (%)	211	13.3	5.5 (0.8)	5.3 (4.1-9.3)	0.5	1.9
LH^h^ (U/L)	206	13	12.9 (13.6)	8.9 (0.3-85)	0.5	1.4
TSH^i^ (mU/L)	176	11.1	2.4 (1.6)	2 (0.1-8.9)	0.6	0.6
PRL^j^ (ng/mL)	140	8.8	24.2 (23.9)	16.3 (0.8-173)	1.4	0.7
E2^k^ (pg/mL)	138	8.7	79.3 (88.2)	46.9 (3.5-516.)	6.4	1.4
FI^l^ (mU/L)	131	8.3	12.9 (12)	9 (1.2-72)	8.2	2.2
P^m^ (ng/mL)	89	5.6	5 (10.1)	0.6 (0.03-47)	12.2	1.1
SHBG^n^ (nmol/L)	72	4.5	84.4 (70.7)	65.5 (14-339.9)	0	0
AMH^o^ (ng/mL)	61	3.8	10.2 (6)	9.2 (0.02-26.)	3.2	1.6
HOMA-IR^p^	57	3.6	2.1 (2.4)	1.6 (0.3-17.7)	3.4	1.7
FT4^q^ (ng/dL)	48	3	1.1 (0.2)	1.1 (0.8-1.7)	4.1	2
17-OHP^r^ (ng/dL)	44	2.8	125.7 (111.8)	79.8 (10-459)	4.5	0
Vit D^s^ (ng/mL)	41	2.6	26.4 (17.3)	24 (4-80)	0	0
FER^t^ (ng/mL)	31	2	37.8 (33)	28.5 (6-167)	0	0
TC^u^ (mg/dL)	30	1.9	219.8 (55.2)	225.5 (105-317)	0	0
LDL^v^ (mg/dL)	27	1.7	132.1 (46.6)	128 (67-290)	0	0
TG^w^ (mg/dL)	25	1.6	129.9 (75.7)	125 (46-300)	0	0
A4^x^ (ng/dL)	23	1.5	349.9 (376.3)	209.1 (1.7-1440)	17.4	0
ALT^y^ (U/L)	23	1.5	90.0 (106.4)	37 (12-412)	0	0
HDL^z^ (mg/dL)	23	1.5	60.7 (18.8)	53 (31-94)	0	0

^a^Total T: total testosterone.

^b^DHEA-S: dehydroepiandrosterone sulfate.

^c^LH/FSH: the ratio of luteinizing hormone to follicle stimulating hormone.

^d^Free T: free testosterone.

^e^FPG: fasting plasma glucose.

^f^FSH: follicle stimulating hormone.

^g^HbA_1c_: glycated hemoglobin.

^h^LH: luteinizing hormone.

^i^TSH: thyroid stimulating hormone.

^j^PRL: prolactin.

^k^E2: estradiol.

^l^FI: fasting insulin.

^m^P: progesterone.

^n^SHBG: sex hormone binding globulin.

^o^AMH: anti-Mullerian hormone.

^p^HOMA-IR: homeostasis model assessment-estimated insulin resistance index.

^q^FT4: free thyroxine.

^r^17-OHP: 17-hydroxyprogesterone.

^s^Vit D: vitamin D.

^t^FER: ferritin.

^u^TC: total cholesterol.

^v^LDL: low-density-lipoprotein cholesterol.

^w^TG: triglycerides.

^x^A4: androstenedione.

^y^ALT: alanine transaminase.

^z^HDL: high-density-lipoprotein cholesterol.

### Comparison to Literature

Table 4 shows the results of the comparison between the PCOS subreddit data and the aggregate of the PCOS literature values. Table 5 shows the individual comparisons between the PCOS subreddit data and each paper used in the aggregation.

**Table 4:** The results of the statistical comparisons between polycystic ovarian syndrome (PCOS) subreddit data and the aggregate of the PCOS literature data.

Result	PCOS subreddit data		Aggregate literature data		Comparison	
	n	Mean (SD)	n	Mean (SD)	Result	*P* value
Age (years)	661	25.5 (5.3)	2843	26 (26.6)	Equivalent	<.001
Total T^a^ (ng/dL)	552	55.9 (37.9)	2378	72.9 (84)	Equivalent	<.001
DHEA-S^b^ (g/dL)	381	396.5 (165.2)	1288	265.5 (293.7)	Literature lower	<.001
BMI (kg/m^2^)	240	24.4 (6.4)	2843	27.4 (28.3)	Equivalent	<.001
Free T^c^ (pg/mL)	226	5.9 (4.4)	911	11.4 (13.7)	Literature higher	<.001
FPG^d^ (mg/dL)	219	94.2 (17.6)	2080	89.7 (90.6)	Equivalent	<.001
FSH^e^ (U/L)	214	5.8 (3.1)	1883	5.9 (6.4)	Equivalent	<.001
LH^f^ (U/L)	206	12.9 (13.6)	1883	8.2 (10.1)	Literature lower	<.001
TSH^g^ (mU/L)	176	2.4 (1.6)	767	2.2 (2.6)	Equivalent	<.001
PRL^h^ (ng/mL)	140	24.2 (23.9)	167	21.4 (26.2)	Equivalent	<.001
FI^i^ (mU/L)	131	12.9 (12)	2080	14.2 (18.6)	Equivalent	<.001
SHBG^j^ (nmol/L)	72	84.4 (70.7)	1423	44.5 (53.8)	Literature lower	<.001
AMH^k^ (ng/mL)	61	10.2 (6)	482	9.9 (12.3)	Equivalent	<.001
HOMA-IR^l^	57	2.1 (2.4)	1135	3 (3.9)	Equivalent	.02

^a^Total T: total testosterone.

^b^DHEA-S: dehydroepiandrosterone sulfate.

^c^Free T: free testosterone.

^d^FPG: fasting plasma glucose.

^e^FSH: follicle stimulating hormone.

^f^LH: luteinizing hormone.

^g^TSH: thyroid stimulating hormone.

^h^PRL: prolactin.

^i^FI: fasting insulin.

^j^SHBG: sex hormone–binding globulin.

^k^AMH: anti-Mullerian hormone.

^l^HOMA-IR: homeostasis model assessment-estimated insulin resistance index.

**Table 5:** The mean of the polycystic ovary syndrome (PCOS) subreddit data set and the PCOS populations in literature with the literature sample numbers in parentheses^a-c^.

	PCOS Subreddit Data		Diamanti-Kandarakis and Panidis [29] (n=634), mean (SD)	Cai et al [30] (n=600), mean (SD)	Tosi et al [31] (n=375), mean (SD)	Paschou et al [32] (n=372), mean (SD)	Sova et al [33] (n=319), mean (SD)	Kumar et al [34] (n=213), mean (SD)	Bahceciet al [35] (n=98), mean (SD)	Homburg et al [36] (n=90), mean (SD)	Pigny et al [37] (n=73), mean (SD)	Wright et al [38] (n=69), mean (SD)
	n	Mean (SD)										
Age (years)	661	25.5 (5.3)	24.3 (5.6)^a^	27.7 (5.2)^a^	23.1 (5.3)^b^	25.5 (6.1)^a^	28.1 (4.3)^c^	27.5 (6.6)^a^	22.4 (3.5)^b^	31.6 (4.4)^c^	29 (4.4)^c^	24.8 (6.2)^a^
Total T^d^ (ng/dL)	552	55.9 (37.9)	79 (28.9)^c^	64.9 (26.8)^a^	—^e^	85.8 (36.5)^c^	46.2 (20.2)^a^	99.7 (89.2)^c^	99 (35)^c^	—	45 (21.6)^b^	50 (25.3)^a^
DHEA-S^f^ (g/dL)	381	396.5 (165.2)	286.3 (119.8)^b^	—	—	288.3 (127.4)^b^	—	163.9 (83.4)^b^	—	—	—	265.3 (118.3)^b^
BMI (kg/m^2^)	240	24.4 (6.4)	26.7 (7.4)^a^	26.4 (5.7)^a^	27.6 (7.1)^c^	27.8 (7)^c^	27.3 (6.3)^c^	36 (9.2)^c^	23.5 (5.9)^a^	24.9 (2.4)^a^	26 (6.1)^a^	21.9 (2.1)^b^
Free T^g^ (pg/mL)	226	5.9 (4.4)	—	13.7 (7.3)^c^	—	—	—	9.2 (5.8)^c^	1.9 (1.1)^b^	—	—	—
FPG^h^ (mg/dL)	219	94.2 (17.6)	96.8 (13.7)^a^	—	85.1 (10)^b^	83.1 (7.8)^b^	91.8 (9)^a^	88 (12.3)^b^	90.5 (9.2)^a^	—	—	80.8 (8.6)^b^
FSH^i^ (U/L)	214	5.8 (3.1)	5.5 (1.7)^a^	6.23 (2.5)^a^	—	—	6.2 (2.1)^a^	—	6.9 (5.6)^a^	5.1 (1.4)^a^	5.5 (1.6)^a^	5.8 (3.2)^a^
LH^j^ (U/L)	206	12.9 (13.6)	7.9 (5.7)^b^	8.8 (6.4)^b^	—	—	6.9 (4.8)^b^	—	7.6 (5)^b^	8.8 (5.2)^b^	6.7 (4)^b^	16.6 (9.7)^a^
TSH^k^ (mU/L)	176	2.4 (1.6)	—	2.3 (1.2)^a^	—	—	—	—	1.7 (1.18)^b^	—	—	2.5 (1)^a^
PRL^l^ (ng/mL)	140	24.2 (23.9)	—	—	—	—	—	—	24.7 (17.1)^a^	—	—	16.6 (9.7)^b^
FI^m^ (mU/L)	131	12.9 (12)	13.3 (14.4)^a^	—	15.7 (11.8)^a^	15.2 (4.8)^a^	11.2 (11.5)^a^	19.7 (14.7)^c^	10.2 (6.2)^a^	—	—	11.3 (6.9)^a^
SHBG^n^ (nmol/L)	72	84.4 (70.7)	38 (20.6)^b^	—	—	31.5 (12.5)^b^	50.9 (27.7)^b^	—	115 (38)^c^	—	—	—
AMH^o^ (ng/mL)	61	10.2 (6)	—	—	—	—	9.3 (6.6)^a^	—	—	10.9 (8.5)^a^	11.4 (8)^a^	—
HOMA-IR^p^	57	2.1 (2.4)	—	—	3.4 (2.8)^c^	3.1 (1.9)^c^	2.6 (2.8)^a^	—	—	—	—	2.3 (1.5)^a^

^a^Statistically equivalent (*P* value from equivalence test was *P*<.05).

^b^Literature value was statistically lower than the subreddit value (*P* value from one-sided upper *t* test was *P*<.05).

^c^Literature value was statistically higher than the subreddit value (*P* value from one-sided lower *t* test was *P*<.05).

^d^Total T: total testosterone.

^e^Not available.

^f^DHEA-S: dehydroepiandrosterone sulfate.

^g^Free T: free testosterone.

^h^FPG: fasting plasma glucose.

^i^FSH: follicle stimulating hormone.

^j^LH: luteinizing hormone.

^k^TSH: thyroid stimulating hormone.

^l^PRL: prolactin.

^m^FI: fasting insulin.

^n^SHBG: sex hormone–binding globulin.

^o^AMH: anti-Mullerian hormone.

^p^HOMA-IR: homeostasis model assessment-estimated insulin resistance index.

## Discussion

### Principal Results

The PCOS subreddit contains dense information that could potentially be used for clinical research. However, the relevance of the information must first be ascertained. Participation within the PCOS subreddit does not require confirmation of a diagnosis and allows anyone with internet access to post. Hence, there is potential for significant biases to arise. In particular, there is likely to be a notable rate of users who do not formally fulfill diagnostic criteria or a bias toward those with limited health care access. However, there is a very large data set of potentially valuable personal accounts on the platform. In particular, the exploration of the PCOS subreddit led to a large number of posts that contained laboratory test results.

The subreddit data set yielded high androgens, high luteinizing hormone (LH), high anti-Mullerian hormone (AMH), and clinical evidence of metabolic issues. Thus, the subreddit users align with the PCOS laboratory result trends described in the literature. Tables 4 and 5 show that alignment was not perfect. However, this is expected, as even any 2 studies with slightly different inclusion and exclusion criteria are unlikely to exclusively achieve statistical equivalence across a range of factors. The broad alignment observed in this study is a positive outcome, as it proves the PCOS subreddit contains data relevant to PCOS. Furthermore, this study allows interesting conclusions to be drawn regarding the PCOS subreddit cohort and why the subreddit data set differs from the literature data sets. The major reasons for differences were likely the subreddit containing more neglected phenotypes, the lack of exclusion criteria in the subreddit, and the discordant exclusion criteria throughout the literature.

As shown in Table 5, the subreddit follicle stimulating hormone (FSH), fasting insulin (FI), and AMH test results were typically either equivalent or at least rarely statistically different from the literature values. The subreddit DHEA-S and LH test results were typically higher than the literature values, while subreddit total T, homeostasis model assessment-estimated insulin resistance index (HOMA-IR), and BMI results were typically lower than or equivalent to the literature values. It is possible that the higher LH values within the subreddit are due to a portion of the laboratory tests being carried out in the mid cycle and luteal phases of menstruation. Similarly, this could imply that the FSH values within the subreddit may be lower in comparison to the literature values when accounting for the different menstrual phases.

Alternatively, a review and meta-analysis found a referral bias in studies toward patients with PCOS who were more obese and had the more classic PCOS phenotype (all Rotterdam criteria present) compared to PCOS populations that had purposefully employed strategies to achieve broad inclusion [11]. As BMI decreases, the likelihood of having a PCOS diagnosis also decreases for individuals who meet the Rotterdam criteria when surveyed [10]. Therefore, the subreddit may have captured more of the phenotypes of PCOS that were difficult to diagnose, such as PCOS with a normal BMI and weaker hyperandrogenism. The abundance of the lower BMI phenotypes would also explain the higher LH, as there is an inverse relationship between BMI and LH in PCOS [3]. It is also possible that people with higher BMIs were less likely to voluntarily include their BMI in the subreddit post due to embarrassment or concern that it would become the focus of the discussion [7].

Similarly, the PCOS subreddit may have attracted people whose PCOS symptoms were more difficult to treat. People with a high HOMA-IR would likely be treated with metformin in many countries, whereas people with a normal HOMA-IR may have come to the subreddit searching for alternative treatments. It is also possible that people with PCOS who had the expected high total T or high HOMA-IR did not post about these results because they understood the implications of these measurements and did not feel the need to seek advice from the subreddit community. A person with the symptoms of PCOS but a low total T value may have been more likely to discuss it in the subreddit due to the common misunderstanding that a person with PCOS must have a high total T [45].

The subreddit fasting plasma glucose (FPG), thyroid stimulating hormone (TSH), and prolactin (PRL) results were greater than or equal to the literature values. However, this was an expected result as some studies excluded people with abnormal BMI [35], obesity [35,38], prediabetes [29,32,38], diabetes [29,31-33,38], high TSH [31,34,38], or high prolactin [30,31,34]. The subreddit data contained no such exclusions. Since PCOS is a metabolic disease with known links to insulin resistance [46], it may be expected that the PCOS subreddit contributors exhibited values more in line with insulin resistance. Age was equitable with some studies and higher or lower than others, thus implying the ages of the PCOS subreddit participants were not outside the range of PCOS cohorts in published studies.

Free testosterone (Free T) was also inconsistent. The larger studies had greater free T values than the PCOS subreddit. This was not unexpected and aligned with the phenomenon of greater total T in the literature. However, a study that excluded all abnormal BMIs when selecting study participants reported a free T value significantly lower than the other literature values and the subreddit value [35]. It has been reported in several PCOS studies that elevated free androgens are strongly associated with elevated BMI [47-49]. Thus, it is logical that the only study to exclude patients with an abnormal BMI is the only one to report a significantly lower free T value.

Sex hormone–binding globulin (SHBG) was inconsistent in a similar way to free T, with the normal BMI study breaking the trend set by the larger studies. The larger studies reported higher SHBG values than the subreddit values, but the normal BMI study found a lower SHBG value. SHBG levels are lower in populations with PCOS [50], but this is especially true in those who also possess a high BMI [47-49]. Since the subreddit BMI was often lower than the literature values, it is expected that the subreddit SHBG would be higher than the literature values that did not exclude based on BMI but lower than the literature values that only took normal-BMI participants.

### Discussion of Methods

A series of statistical equivalence tests were carried out to compare the important values across Reddit data and typical studies. The comparisons made in this paper were exploratory, and interpretation of Table 5 allows the subreddit values to be placed in the context of published literature values. No particular comparison was considered important in isolation; rather, all comparisons were considered together. Since this research was not intended to capture significance in a single isolated statistical test, undertaking statistical testing to account for multiple comparisons was unnecessarily conservative. Furthermore, *P*<.05 was suitable to allow rejection of the null hypothesis for all tests.

The use of a CNN to select posts likely to contain laboratory test results reduced manual processing time. However, there remains the possibility that some posts containing laboratory test information were overlooked due to uncommon phrasing that was not recognized by the automated algorithm. This would only affect results if the rate of omission was high and there was a bias in the reported values of the omitted posts. The CNN’s overall accuracy of 98% (89/91) on the testing data implies that a high rate of omission is extremely unlikely.

While it is important to carefully select the study cohorts to avoid obscuring the effect the study seeks to capture, it is also important to consider the bias ubiquitous with such exclusion criteria. By removing people with higher BMIs, as some studies do [35,38], up to an estimated 61% [51] of the PCOS population may be ignored. Similarly, by removing people with high TSH, up to 34% [30] of the PCOS population may be overlooked. The method of data collection used here allowed for a more complete exploration of PCOS and conditions that may be mistaken for it.

However, this data collection method was not without its problems. The data set was limited to English speakers with internet access and knowledge of the subreddit’s existence. It was also biased toward people with a desire to post in the subreddit, such as people without reliable, affordable, or satisfying clinical care. However, it must be noted that typical observational studies have similar biases. In particular, most studies involving laboratory tests require volunteers to actively engage in and participate in clinical research (Table 2). Furthermore, typical studies often recruit from clinics, thus requiring primary patient attendance, which is linked to affluence or the intensity of symptoms. Thus, the demands of compliance in typical studies are likely to lead to biases different, but no less important, than the biases observed in the PCOS subreddit cohort. Furthermore, the biases in the subreddit represent an important, perhaps overlooked, sector of patients with PCOS that requires investigation.

Additionally, the way users presented their results often led to ambiguity and uncertain values in the data set. The results for progesterone (P), estradiol (E2), and Vitamin D had high proportions of uncertainty due to large and overlapping ranges for different common units. Another common cause of uncertainty was determining if an insulin or glucose test was fasting when that was not stated. Differentiating between free and total testosterone without units was also difficult when the given value was small, as total T in nmol/L and free T in pg/mL both allow for small values. Free thyroxine (FT4) had high uncertainty due to difficulty distinguishing between total thyroxine and FT4 when the full name was not stated. However, very few test results had over 5% uncertainty.

It was difficult to determine whether people posting on the PCOS subreddit were on medications that would impact their laboratory test results. Additionally, as anyone can post on the subreddit, some of the posters would not have clinically confirmed PCOS. Developments in text interpretation software may enable the isolation of values from people who have not been diagnosed or people on certain medications. However, without this step, this study shows the PCOS subreddit contained data that was aligned with what would be expected from a PCOS data set.

### The PCOS Subreddit

Due to the inclusive nature of the web-based forum, a reasonable expectation would have been average laboratory test results that measured between the levels of PCOS dysregulation in the literature and normal results. While this was the case for many of the results, other results unexpectedly showed more extreme PCOS dysregulation than the literature values. This was likely also a result of the more inclusive nature of the subreddit and the tendency of published research to exclude people with multiple conditions. Regardless of the differences between the PCOS subreddit and the literature values, the comparisons between them have validated the usefulness of the subreddit data set. They also allow an indication of which dysregulations seem most consistent across PCOS. The subreddit values of FSH, FI, and AMH were almost entirely equivalent to the PCOS literature values, implying these test results are perhaps more consistent features of PCOS dysregulation across the most PCOS phenotypes when compared to other results.

The PCOS subreddit provides a uniquely inclusive look at a variety of people presenting with PCOS-like symptoms. Furthermore, analysis of the subreddit could be made quickly, inexpensively, and with large cohort populations. Thus, the PCOS subreddit may provide data that can be analyzed in specific clinical research ventures. The unprompted and self-reported nature of the data set creates a unique situation where people discuss only what they consider relevant rather than providing a complete overview of their symptoms. This leads to a data set that places more importance on what people having PCOS perceive as the most problematic aspects of the syndrome.

### The Potential of Medical Internet Forum Data Sets

There are several benefits to extracting data for clinical research from freely available internet forums targeting certain conditions. The individuals posting in internet forums will likely represent a different, more diverse subpopulation than those participating in traditional clinical research. It is likely that the conditions with the most dissatisfied patients will have the most active forums, making it easy for researchers to target the groups with unmet clinical needs. The data is also very abundant and readily available at a low direct cost. This data availability allows a broad research lens for certain conditions. However, due to the voluntary participation in the forum, it is likely that some participants do not actually have the condition of interest, and ultimately add noise to the data. In contrast, traditional clinical research can ensure that individuals in the population studied have the condition of interest due to the strict screening processes. However, traditional clinical research is resource intensive and must choose between a narrow scope and low numbers. There will be a point when statistical inference from the large numbers in the forum overcomes the poor signal-to-noise ratio.

While machine learning is currently undergoing swift development, the authors do not believe it is currently reliable enough to process large amounts of data for clinical research without supervision. Creating supervised machine learning algorithms is arduous as it requires manual processing of large subsets of the data. It is likely that in the future, machine learning will advance to the point where the data can be processed both easily and accurately, but even in that case, there will be limitations. In particular, care must be taken to ensure the data source has well-understood biases to ensure interpretation of the outcomes is clinically meaningful. It is easy to misuse machine learning without careful consideration of how it is being trained [52]. Overall, traditional clinical research and automated forum data extraction both yield very distinct but uniquely useful types of data.

### Conclusions

Extracting PCOS subreddit laboratory results using a machine learning approach produced a large data set of demographic and laboratory values obtained from individuals with self-reported PCOS displaying a range of clinical phenotypes. The subreddit results were broadly consistent with a PCOS population. Comparisons of reported subreddit laboratory data with those of recently published observational studies for patients with PCOS showed equivalence across some of these values. The most equivalent results across the PCOS literature data and the subreddit data were FSH, FI, and AMH, opening the possibility that these PCOS dysregulations are less dependent on PCOS clinical phenotypes than others. Even subreddit results that were not equivalent to the literature values were consistent with PCOS dysregulations. Due to the large number of posts, the subreddit data set could potentially offer an avenue to analyze the differing biochemical phenotypes of PCOS, such as those with elevated DHEA-S rather than elevated total T. Thus, this research validates the approach of extracting PCOS subreddit data for the purpose of undertaking exploratory clinical research on a large number of people with PCOS.

## Appendix

Multimedia Appendix 1 Details on the machine learning used to identify posts likely to contain laboratory test results.

Multimedia Appendix 2 Unit considerations used to assess if a unit is wrong or a value is nonsense.

Multimedia Appendix 3 All unit conversions made in the PCOS subreddit laboratory test results analysis.

Multimedia Appendix 4 A summary of the results given for the cycle phase at the time of laboratory tests.

Multimedia Appendix 5 Age and BMI in PCOS subreddit laboratory test result population (when available).

## Abbreviations

AHRECS: Australasian Human Research Ethics Consultancy Services
AMH: anti-Mullerian hormone
CNN: convolutional neural network
DHEA-S: dehydroepiandrosterone sulfate
E2: estradiol
FI: fasting insulin
FPG: fasting plasma glucose
Free T: free testosterone
FSH: follicle stimulating hormone
FT4: free thyroxine
HOMA-IR: homeostasis model assessment-estimated insulin resistance index
LH: luteinizing hormone
P: progesterone
PCOS: polycystic ovary syndrome
PRL: prolactin
SHBG: sex hormone–binding globulin
TOST: two one sided t test
Total T: total testosterone
TSH: thyroid stimulating hormone

## Data Availability

All the raw data used in this study is publicly available on the PCOS subreddit [16] and can be easily accessed through Pushshift [23]. The authors plan to release the gathered laboratory data within 24 months of this publication, along with the other processed PCOS data sets that are emerging from this research.
