# Supplementary material for: Comparing Literature- and Subreddit-Derived Laboratory Values in Polycystic Ovary Syndrome (PCOS): Validation of Clinical Data Posted on PCOS Reddit Forums
Source: JMIR Form Res. 2023 Aug 25;7:e44810. doi: 10.2196/44810 (PMC10492173; doi:10.2196/44810)
Supplement: Multimedia Appendix 1 [file formative_v7i1e44810_app1.docx]

## Multimedia Appendix 1

The CNN (convolutional neural network) structure closely followed that of Kim [25] but using the symMSAF activation function [26] after the convolutional layer. There were 4 filter sizes used to look at 2, 5, 10, and 15 words at a time. There were 5 filters of each size, leading to a total of 20 filters.

There was a substantial class imbalance withing the 5,000 labelled posts, with only 163 labelled as containing laboratory test results. To account for that, only 200 posts not containing laboratory test results were selected to make up the training and testing data with the posts that did contain laboratory test results. These 363 posts were shuffled and split into a training dataset with 80% of the posts and a testing dataset with the remaining 20%.

The CNN was trained with a batch size of 10 until the average loss stayed within 1e-3 of the previous loss or the loss from the testing data began to increase. The loss function was also weighted towards the laboratory test results present class with a ratio of 3:1. After training, the CNN was tested using the allocated testing dataset and the posts without laboratory test results that was not included in the initial selecting of the training and testing dataset.
