# Supplementary material for: Comparing Literature- and Subreddit-Derived Laboratory Values in Polycystic Ovary Syndrome (PCOS): Validation of Clinical Data Posted on PCOS Reddit Forums
Source: JMIR Form Res. 2023 Aug 25;7:e44810. doi: 10.2196/44810 (PMC10492173; doi:10.2196/44810)
Supplement: Multimedia Appendix 2 [file formative_v7i1e44810_app2.docx]

## Multimedia Appendix 2

**Table S**1. Unit considerations used to assess if a unit is wrong or a value is nonsense.

| **Test Result** | **Healthy Reference Ranges in Common Units** | | | **Unit Considerations** |
| --- | --- | --- | --- | --- |
| Total T | 20-75 ng/dL | 0.7-2.6 nmol/L | 0.15-0.7 ng/mL | If value < 5, then likely not ng/dL |
| DHEA-S | 59-328 $\mu$g/dL | 1.6-8.9 $\mu$mol/L | -- | If value < 15, then likely $\mu$mol/L |
| FT | - - 1. pg/mL | 0-1.15 ng/dL | 0-40 pmol/L | If value > 20, then likely pmol/L |
| FPG | 70-99 mg/dL | 3.9-5.5 mmol/L | -- | If value < 10, then likely mmol/L  If value between 25-45, then likely nonsense |
| HbA1c | 4-5.6 % | 20-37.6 mmol/mol | -- | If value > 15, then likely mmol/mol  If value < 3, then likely nonsense |
| PRL | 2-28 ng/mL | 50-650 mU/L | -- | If value < 30, then likely ng/mL |
| FI | < 25 mU/L | < 174 pmol/L | -- | If value > 300, then likely pmol/L |
| 17-OHP | < 200 ng/dL | < 2 ng/mL | 6.06 nmol/L | If value > 100, then likely ng/dL |
| A4 | 29-329 ng/dL | 1-11.5 nmol/L | -- | If value > 200, then likely ng/dL |
