# Supplementary material for: Comparing Literature- and Subreddit-Derived Laboratory Values in Polycystic Ovary Syndrome (PCOS): Validation of Clinical Data Posted on PCOS Reddit Forums
Source: JMIR Form Res. 2023 Aug 25;7:e44810. doi: 10.2196/44810 (PMC10492173; doi:10.2196/44810)
Supplement: Multimedia Appendix 3 [file formative_v7i1e44810_app3.docx]

## Multimedia Appendix 3

**Table S2**. All unit conversions made in the PCOS subreddit laboratory test results analysis.

| **Test Result** | **Unit To Convert To** | **Conversions** | |
| --- | --- | --- | --- |
| Total T | ng/dL | 1 nmol/L * 28.85 | 1 ng/mL * 100 |
| DHEA-S | $\mu$g/dL | 1 $\mu$mol/L * 37.0 | -- |
| FT | pg/mL | 1 ng/dL * 10.0 | 1 pmol/L * 0.2885 |
| FPG | mg/dL | 1 mmol/L * 18.0 | -- |
| HbA1c | % | [(1 mmol/mol – 9.0) / 11.0] + 3.0 | -- |
| PRL | ng/mL | 1 mU/L * 0.047 | -- |
| E2 | pg/mL | 1 pmol/L * 0.272 | -- |
| FI | mU/L | 1 pmol/L * 0.143988 | -- |
| P | ng/mL | 1 nmol/L * 0.3145 | -- |
| AMH | ng/mL | 1 pmol/L * 0.14 | -- |
| FT4 | ng/dL | 1 pmol/L * 0.0775 | -- |
| 17-OHP | ng/dL | 1 ng/mL * 100.0 | 1 nmol/L * 33.046 |
| Vit D | ng/mL | 1 nmol/L * 0.4 | -- |
| TC, LDL, HDL | mg/dL | 1 mmol/L * 38.6 | -- |
| TG | mg/dL | 1 mmol/L * 88.5 | -- |
| A4 | ng/dL | 1 nmol/L * 28.65 | -- |
