# Supplementary material for: Comparing Literature- and Subreddit-Derived Laboratory Values in Polycystic Ovary Syndrome (PCOS): Validation of Clinical Data Posted on PCOS Reddit Forums
Source: JMIR Form Res. 2023 Aug 25;7:e44810. doi: 10.2196/44810 (PMC10492173; doi:10.2196/44810)
Supplement: Multimedia Appendix 4 [file formative_v7i1e44810_app4.docx]

**Table S3**. A summary of the results given for the cycle phase at the time of laboratory tests.

| Cycle phase | Proportion of total phase results (N=73), n (%) |
| --- | --- |
| Follicular | 30 (41) |
| Mid cycle | 13 (18) |
| Luteal | 30 (41) |
