# Supplementary material for: Comparing Literature- and Subreddit-Derived Laboratory Values in Polycystic Ovary Syndrome (PCOS): Validation of Clinical Data Posted on PCOS Reddit Forums
Source: JMIR Form Res. 2023 Aug 25;7:e44810. doi: 10.2196/44810 (PMC10492173; doi:10.2196/44810)
Supplement: Multimedia Appendix 5 [file formative_v7i1e44810_app5.docx]

## Multimedia Appendix 5

| 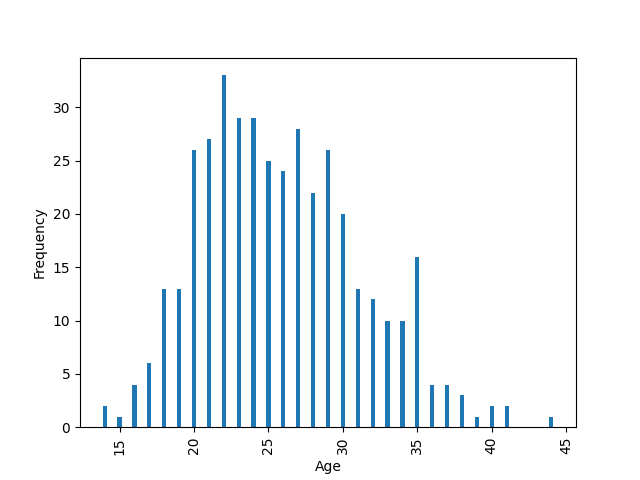 | 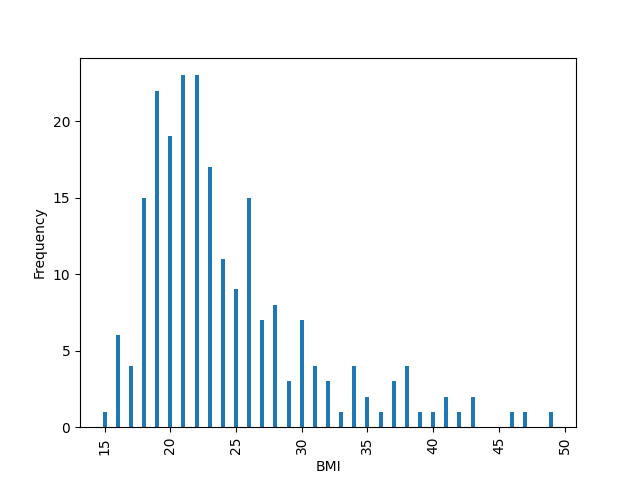 |
| --- | --- |

**Figure S1**. Age and BMI in PCOS subreddit laboratory test result population (when available).
